# BRUCE silencing leads to axonal dystrophy by repressing autophagosome-lysosome fusion in Alzheimer’s disease

**DOI:** 10.1038/s41398-021-01427-2

**Published:** 2021-08-05

**Authors:** Lu Zhang, Yu Fang, Xinyu Zhao, Yake Zheng, Yunqing Ma, Shuang Li, Zhi Huang, Lihao Li

**Affiliations:** 1 Department of Neurology, The First Affiliated Hospital of Zhengzhou University, Zhengzhou, 450052 P.R. China; 2 ICU, The First Affiliated Hospital of Zhengzhou University, Zhengzhou, 450052 P.R. China

**Keywords:** Neuroscience, Biomarkers

## Abstract

Axonal dystrophy is a swollen and tortuous neuronal process that contributes to synaptic alterations occurring in Alzheimer’s disease (AD). Previous study identified that brain-derived neurotrophic factor (BDNF) binds to tropomyosin-related kinase B (TrkB) at the axon terminal and then the signal is propagated along the axon to the cell body and affects neuronal function through retrograde transport. Therefore, this study was designed to identify a microRNA (miRNA) that alters related components of the transport machinery to affect BDNF retrograde signaling deficits in AD. Hippocampus tissues were isolated from APP/PS1 transgenic (AD-model) mice and C57BL/6J wild-type mice and subject to nicotinamide adenine dinucleotide phosphate and immunohistochemical staining. Autophagosome-lysosome fusion and nuclear translocation of BDNF was detected using immunofluorescence in HT22 cells. The interaction among miR-204, BIR repeat containing ubiquitin-conjugating enzyme (BRUCE) and Syntaxin 17 (STX17) was investigated using dual luciferase reporter gene assay and co-immunoprecipitation assay. The expression of relevant genes and proteins were determined by RT-qPCR and Western blot analysis. Knockdown of STX17 or BRUCE inhibited autophagosome–lysosome fusion and impacted axon growth in HT22 cells. STX17 immunoprecipitating with BRUCE and co-localization of them demonstrated BRUCE interacted with STX17. BRUCE was the target of miR-204, and partial loss of miR-204 by inhibitor promoted autophagosome–lysosome fusion to prevent axon dystrophy and accumulated BDNF nuclear translocation to rescue BDNF/TrkB signaling deficits in HT22 cells. The overall results demonstrated that inhibition of miR-204 prevents axonal dystrophy by blocking BRUCE interaction with STX17, which unraveled potential novel therapeutic targets for delaying AD.

## Introduction

Dystrophic axons (axonal dystrophy), also known as dystrophic neuritis or neuroaxonal dystrophy, are considered one of the major neuropathological features of Alzheimer’s disease (AD)^1^. It has been revealed that dystrophic axons contained the accumulated tubulovesicular structures, filamentous materials, and swollen edematous mitochondria with a degenerated inner membrane that are localized in the central nervous system^2^. The two major diseases of dystrophic axons are pantothenate kinase-associated neurodegeneration and infantile neuroaxonal dystrophy^3^. Previous studies have reported that phosphodiesterase inhibitors can reverse axonal dystrophy in mice with Friedreich’s ataxia, and a brain-penetrant triazolopyrimidine alleviates the axonal dysfunction in tauopathy mice^4,5^. Nevertheless, the exact mechanism of the dystrophic axons in AD remains to be elucidated.

Interestingly, it has been revealed that the neurotrophins function as a crucial regulator of axon regeneration, especially brain-derived neurotrophic factor (BDNF)^6^. Neurotrophins are endogenous peptides that enhance the survival and maintenance of neurons. The member of neurotrophins, BDNF, has been revealed to exert neuroprotective effects in different animal models of neurodegeneration^7^. In addition, previous researches have reported that retrograde transport of BDNF-activated tropomyosin-related kinase B (TrkB) receptors was involved in the process of autophagosomes, which improves the neuronal complexity and preventing neurodegeneration in vivo, and the BDNF expression was modulated by sortilin-mediated trafficking and lysosomal degradation^8,9^. Lysosomes have been found to be significant in maintaining the integrity of neuronal function, and mutations in genes inducing lysosome formation, transport, and activity related to neurodegenerative disorders^10^. In addition, the inhibitor of apoptosis family of proteins (IAP) BIR repeat-containing ubiquitin-conjugating enzyme (BRUCE) was reported to modulate the fusion of autophagosome and lysosome, and also interacted with Syntaxin 17 (STX17), an important mediator in autophagosome–lysosome fusion^11^. The function of STX17 as a crucial protein during the fusion of autophagosome and lysosome in non-cardiomyocytes has also been demonstrated previously^12^. Furthermore, the upregulation of microRNA-204 (miR-204) in the distal axons of sympathetic neurons has been identified^13^ and was involved in the regulation of transcription and informatics-identified pathways in the processes of neurogenesis and axon guidance^14^. Interestingly, miR-204 has been proved to target BDNF in tumor progression^15^. Therefore, from the findings above, it was hypothesized that the miR-204/BRUCE/STX17/BDNF axis may affect the development of dystrophic axons in AD by regulating the fusion of autophagosome and lysosome. Hence, the present study aimed to test this hypothesis and explore the underlying mechanism of axonal dystrophy in AD, which provides insight into the therapeutic targets for the treatment for AD.

## Methods and materials

### Ethics statement

All of the experiments involving rodents in this study were approved by the Committee on Animal Care and Use from the First Affiliated Hospital of Zhengzhou University. All animal experiments were performed in strict accordance with the recommendations in the Guide for the Care and Use of Laboratory Animals of the National Institutes of Health and conform to relevant national provisions.

### Study subjects

The male APP_SWE_/PS1_ΔE9_ transgenic mice (age: 12 weeks) and C57BL/6J wild-type (Wt) mice (age: 12 weeks) that used in this study were purchased from HFK Biotechnology Co., Ltd. (Beijing, China). The mice were reared in the cage with polypropylene (34 × 22 × 15 cm) under standard conditions (room temperature of 22.5 ± 1 °C, humidity of 50 ± 2% and 12 h of light and dark cycle) with free access to food and water. For tissue analysis, all mice were euthanized by CO_2_ asphyxiation, and the hippocampus was isolated from the mice brain, sectioned into 4 μm, and stained by nicotinamide adenine dinucleotide phosphate (NADPH-d). Subsequently, the sections were observed and analyzed under a microscope.

The procedures of NADPH-d staining were as follows: frozen sections were pre-incubated for 5–10 min in Tris-buffer solution containing Triton X-100, and incubated for 1–1.5 h in Tris-buffer solution containing Triton X-100, NADPH-d, and nitroblue tetrazolium. After the termination of the reaction, the sections were cleared and mounted.

### Immunohistochemistry (IHC)

Sectioned hippocampus tissues were incubated with diluted mouse anti-BDNF antibody (5 µg/mL, ab203573, Abcam, Cambridge, UK) for 16 h at 4 °C and with horseradish peroxidase (HRP)-labeled rabbit anti-mouse immunoglobulin G (IgG) (ab6728, Abcam) for 2 h. Subsequently, the sections were stained for 5 min with diaminobenzidine (AR1000; Wuhan Boster Biological Technology Co., Ltd., Wuhan, Hubei, China), then observed and photographed under the optical microscope.

### Harvest of primary hippocampal neurons HT22

In brief, primary culture was obtained from mouse hippocampus on the 18th embryonic day, dissected under sterile condition, and detached with 0.125% trypsin at 37 °C for 20 min. Then, complete medium (Neurobasal2, Gibco, Grand Island, NY, USA) containing 2% B27 (Gibco), 2 mM alanyl-glutamine (Gibco), 10% fetal bovine serum (FBS), and 1 mM penicillin/streptomycin (Sigma, St. Louis, MO, USA) were applied to terminate the detachment. Tissues were washed by FBS-free complete medium and dissociated using a plastic pipette. Neurons were spread on a petri dish with a glass bottom at a density of 0.25–1 × 10^5^ cells/mL and allowed to stand for 2 h in the incubator for attachment. Then, a serum-free medium was added until the volume reached 2/3. Primary hippocampal neurons HT22 (CL-0595; Procell Life Science & Technology Co., Ltd., Hubei, China; https://www.procell.com.cn/view/4535.html) were used for in vitro experiments in this study. HT22 cells were cultured in the Dulbecco’s modified Eagle’s medium containing 10% FBS, 2 mM L-alanyl-L-glutamate, 0.24% hydroxyethyl piperazine ethane-sulfonic acid (HEPES), 0.375% sodium bicarbonate, 100 U/mL penicillin, and 100 mg/mL streptomycin.

### Cell treatment

HT22 cells were detached with trypsin, counted, seeded in a 6-well plate at a density of 5 × 10^5^ cells/well, and allowed to stand overnight for attachment. Upon reaching 80% confluence, subsequent experiments were performed. HT22 cells were washed 3 times with Dulbecco’s phosphate-buffered saline (DBPS) and cultured in starvation medium (40 mM NaCl, 1 mM CaCl_2_, 1 mM MgCl_2_, 5 mM glucose, and 20 mM HEPES, pH 7.4) at 37 °C in order to allow the cells to achieve a starvation state.

To block the autophagy flux, part of the cells were interfered with using Bafilomycin A1 (Abmole Bioscience Inc., CA, USA) at concentrations of 10, 20, and 50 nM, respectively. The autophagosome staining was performed using an MDC kit (P6659-20 μg Recombinant Human MDC/CCL22 20 μg, Beyotime Biotechnology Co., Ltd., Shanghai, China), and lysosome staining was performed using Lys-tracker-Red kit [C1046 Lyso-Tracker Red (lysosome red fluorescent probe) 50 μL, Beyotime]. After 24 h of treatment, the cells were photographed and observed under microscope^16^.

For cell transfection, the adenoviruses pAd/CMV/V5-DEST and pAd/BLOCK-iT-DEST RNAi Gateway Vector were purchased from BioVector NTCC (Beijing, China). The overexpression of BRUCE (oe-BRUCE) and short hairpin RNA against STX17 (sh-STX17) and BRUCE (sh-BRUCE), as well as corresponding negative control (NC) (oe-NC and sh-NC), were purchased from GenePharma Co., Ltd. (Shanghai, China). Also, the miR-204 mimic/inhibitor was purchased from GenePharma. The cell transfection was conducted in accordance with the manufacturer’s instructions of Lipofectamine 2000 (Invitrogen, Carlsbad, CA, USA). In addition, the construction of HT22 cell line with dominant-negative STX17 mutant (Mut) GFP-STX17△NTD or full-length STX17 GFP-STX17FL overexpression was performed based on a previous study^12^.

### Western blot analysis

HT22 cells or mouse hippocampus tissues were lysed for total protein extraction. For nuclear protein extraction, HT22 cells or mouse hippocampus tissues were lysed and centrifuged at 4000 rpm to collect the precipitate. Then, the precipitate was resuspended and centrifuged at 13,000 rpm for 7 min. Total or nuclear protein (40 μg of protein per lane) was separated by 10% sodium dodecyl sulfate (SDS)-polyacrylamide gel electrophoresis and transferred onto the polyvinylidene fluoride membrane (Millipore, Billerica, MA, USA). Subsequently, the membrane was blocked with 5% skimmed milk powder at room temperature for 2 h, and then incubated at 4 °C overnight with following primary antibodies: rabbit anti-BDNF (1:1000, ab108319), phosphorylated TrkB (p-TrkB)/TrkB (1:500, 4621/4606), rabbit anti-p-cAMP-response element-binding protein (CREB) (1:50, ab32096), rabbit anti-CREB (1:1000, ab32515), rabbit anti-STX17 (1:1000, ab229646), and rabbit anti-BRUCE (1:5000, ab19609). All the antibodies above were purchased from Abcam except p-TrkB/TrkB (Cell Signaling Technology, Beverly, MA, USA). After overnight incubation, the membrane was incubated with secondary antibody HRP-labeled anti-rabbit IgG (1:20,000, Thermo Fisher Scientific, MA, USA). Glyceraldehyde-3-phosphate dehydrogenase (GAPDH) or LaminA served as an internal reference, and the protein bands were observed using an electrochemiluminescence kit (CoWin Biosciences, Beijing, China).

### Reverse transcription-quantitative polymerase chain reaction (RT-qPCR)

The total RNA was extracted from mouse hippocampus tissues or HT22 cells. Reverse transcription was done according to the manufacturer’s instructions of SuperScript III First-Strand Synthesis SuperMix. Then, the expression of miR-204 was detected using TaqMan miRNA assay (Ambion, Austin, TX, USA) with U6 used as the internal reference, while the mRNA expression of other genes was examined using PrimeScript RT-PCR kits (Roche, Basel, Switzerland) with GAPDH used as the internal reference. All primers were synthesized by Takara Biotechnology Ltd. (Dalian, Liaoning, China) (Table S1). The fold changes of target genes between the experimental group and the control group were calculated using the 2^−^^ΔΔCt^ method.

### Immunofluorescence staining

The cells grown on slides were fixed with 4% paraformaldehyde for 15 min and blocked with 1× phosphate buffer saline supplemented with 3 mg/mL bovine serum albumin, 100 mM glycine, and 0.25% Triton X-100 for 30 min. Subsequently, the slides were incubated with primary antibody rabbit anti-BDNF (1:500, ab108319), rabbit anti-light chain 3II (LC3II) (1:1000, ab48394), mouse anti-Tau (Tau-13, 1:500, Santa Cruz Biotechnology, Santa Cruz, CA, USA) and rabbit anti-BRUCE (5 µg/mL, ab19609) at 4 °C, followed by culture with the combination of the fluorophore and Alexa Fluor^®^ 647 secondary antibody (1:200, ab150075) at room temperature for 1 h. All the antibodies used above were provided by Abcam except Tau. Then, the cell slides were added with 4′ 6-diamidino-2-phenylindole for nucleus staining, then immersed in distilled water, dried and observed under a fluorescence microscope (Zeiss, Thornwood, NY) or FV-1000 confocal microscope.

For morphological analysis, the cells were fixed with 4% paraformaldehyde for 15 min and incubated with tau antibody (1:200, ab64193, Abcam) for 1 h, followed by another incubation with fluorophore combined with Alexa Fluor^®^ 647 secondary antibody (1:200, ab150075) for 1 h. The cells were then observed under the fluorescence microscope, and the length of the main axon in each cell was measured by five independent experiments.

### Dual-luciferase reporter gene assay

The synthetic BRUCE-3′ untranslated region (UTR) gene fragments were introduced into pMIR-reporter (Beijing Huayueyang Biotechnology Co., Ltd., Beijing, China) using endonucleases SpeI and Hind III. With the complementary sequence Mut site of seed sequence designed in BRUCE Wt, the targeted fragments after restriction endonuclease digestion were inserted into the pMIR-reporter plasmid.

The correctly sequenced luciferase reporter plasmids, Wt and Mut were co-transfected with miR-204 and transfected into HEK-293T cells (CRL-1415, Shanghai Xin Yu Biotech Co., Ltd., Shanghai, China). After 48 h, the cells were collected and lysed. Lastly, the luciferase activity was determined using the Luciferase Detection Kit (RG005, Beyotime) on Glomax20/20 Luminometer Fluorescence Detector (Promega, Madison, WI, USA).

### Co-immunoprecipitation (Co-IP) assay

HT22 cell lysates were collected, and one part was used as input. Every 500 μg of lysate was incubated with antibody and Dynabeads Protein G (Invitrogen, Carlsbad, CA, USA) at 4 °C for protein complexes to pull down. The IgG (Santa Cruz Biotechnology, Inc., Santa Cruz, CA, USA) served as the NC. Subsequently, the beads were washed with cold lysis buffer and boiled in 1× SDS loading buffer for 15 min to elute proteins, followed by Western blot analysis.

### Statistical analysis

All data were statistically analyzed using SPSS 21.0 statistical software (IBM Corp., Armonk, NY, USA). Measurement data were expressed as mean ± standard deviation. Data following normal distribution and homogeneity of variance were compared by unpaired *t*-test between two groups. Data among multiple groups were compared by one-way analysis of variance followed by Tukey’s post hoc test. The repeated-measures ANOVA was applied for data among multiple groups at different time points, followed by the Bonferroni post hoc test. *p* < 0.05 was considered as statistically significant.

## Results

### BDNF/TrkB signaling is implicated in AD mice

**Fig. 1:**
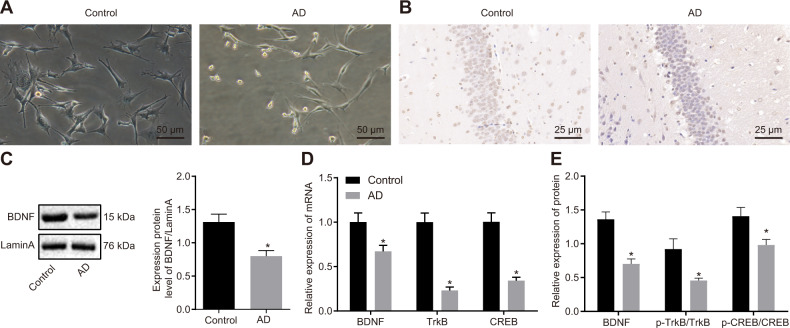
BDNF/TrkB signaling is associated with dystrophic axons in APP/PS1 transgenic mice. The mice used for the following detection were APP_SWE_/PS1_ΔE9_ transgenic AD mice (*n* = 6) and C57BL/6J Wt mice (*n* = 6). **A** The length and morphological structure of axons in the hippocampus of AD mice and Wt mice observed under an optical microscope (200×). **B** The intranuclear BDNF expression in the hippocampus tissues of mice examined by IHC (400×). **C** The expression of BDNF in the nucleus of the hippocampus tissues of mice detected by Western blot analysis, *p < 0.05 compared with that of Wt mice. **D** The mRNA expression of BDNF, TrkB, and CREB in the hippocampus tissues of AD mice and Wt mice detected by RT-qPCR, *p < 0.05 com*p*ared with that of Wt mice. **E** The protein expression of BDNF, p-TrkB/TrkB, and p-CREB/CREB in the hippocampus tissues of AD mice and Wt mice examined by Western blot analysis, *p < 0.05 compared with that of Wt mice. The experiment was repeated three times independently.

The length and morphological structure of axons in the hippocampus of APP_SWE_/PS1_ΔE9_ transgenic AD mice and C57BL/6J Wt mice were analyzed, as shown in Fig. 1A. Compared with the Wt mice, AD mice exhibited shorter and swollen axons. Then, IHC was conducted to examine the BDNF expression in the hippocampus tissues. Results revealed that the BDNF expression in the nucleus was sharply decreased in the hippocampus tissues of AD mice relative to Wt mice (Fig. 1B). Meanwhile, it was further proved by Western blot analysis that BDNF expression in the nucleus was markedly lower in AD mice when compared to Wt mice (Fig. 1C). In addition, RT-qPCR and Western blot analyses were performed to detect the mRNA and protein expression of BDNF/TrkB signaling pathway-related factors (BDNF, p-TrkB/TrkB, and p-CREB/CREB). Results demonstrated that the mRNA and protein expression of BDNF, p-TrkB/TrkB, and p-CREB/CREB was notably reduced in the hippocampus tissues of AD mice when compared to Wt mice (Fig. 1D, E). Hence, these finding indicates that BDNF/TrkB signaling is affected in AD mice.

### Autophagosome–lysosome fusion mediates BDNF nuclear transport in HT22 cells

**Fig. 2:**
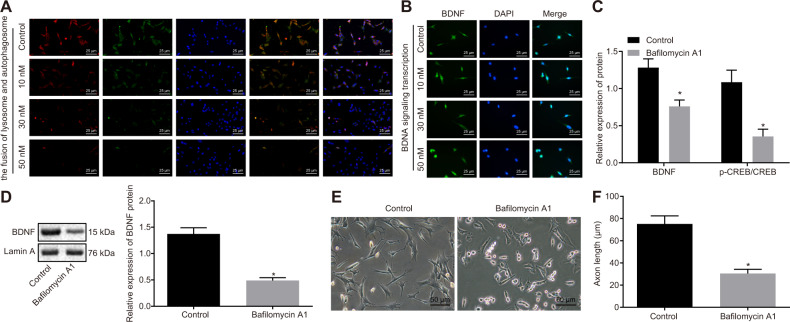
Autophagosome–lysosome fusion mediates BDNF nuclear transport and axonal dystrophy. **A** The fusion of autophagosome and lysosome detected by immunofluorescence staining (400×). **B** The BDNF signaling nuclear import detected by immunofluorescence staining (400×). **C** The protein expression of BDNF and p-CREB/CREB determined by Western blot analysis, *p < 0.05 compared with that of cells without treatment. **D** The protein expression of BDNF in the nucleus measured by Western blot analysis, *p < 0.05 compared with cells without treatment. **E** The morphological changes of axons of HT22 cells after interference with 30 nM Bafilomycin A1 for 24 h (200×). **F** The immunofluorescence staining of axons of HT22 cells after interference with 30 nM of Bafilomycin A1 for 24 h, Tau as a marker for axons, with the axon length recorded; *p < 0.05 compared with cells without treatment. The experiment was repeated three times independently.

In order to further explore the mechanism underlying nuclear transport of BDNF, HT22 cells were initially treated with starvation culture and then added with proton pump inhibitor Bafilomycin A1 to inhibit the fusion of autophagosome and lysosome^17^. The cells were interfered with using Bafilomycin A1 at concentrations of 10, 20, and 50 nM, respectively for 24 h, the cells were then photographed, recorded and immunofluorescence staining was performed subsequently. Results demonstrated that the autophagosome-lysosome fusion reduced with the increasing concentration of Bafilomycin A1, and the autophagosome-lysosome fusion was inhibited most dramatically after the interference using 50 nM Bafilomycin A1, as presented in Fig. 2A. Also, the BDNF signaling nuclear import was gradually decreased with the increasing concentration of Bafilomycin A1, and the significant changes of BDNF signaling nuclear import were detected after treatment of 50 nM Bafilomycin A1 (Fig. 2B). In addition, results from Western blot analysis showed that when compared with cells without treatment, the cells treated with Bafilomycin A1 presented an obvious decrease in protein expression of BDNF/TrkB signaling pathway-related factors and BDNF protein expression in the nucleus (Fig. 2C, D). After 24-h treatment with Bafilomycin A1, the axon length and cell body of HT22 cells were obviously reduced upon treatment of 30 nM Bafilomycin A1 (Fig. 2E, F and Fig. S1A). In a word, autophagosomes–lysosomes fusion regulated BDNF nuclear transport and further led to axonal dystrophy.

### Knockdown of STX17 inhibits the autophagosome–lysosome fusion and affects the axon growth in HT22 cells

**Fig. 3:**
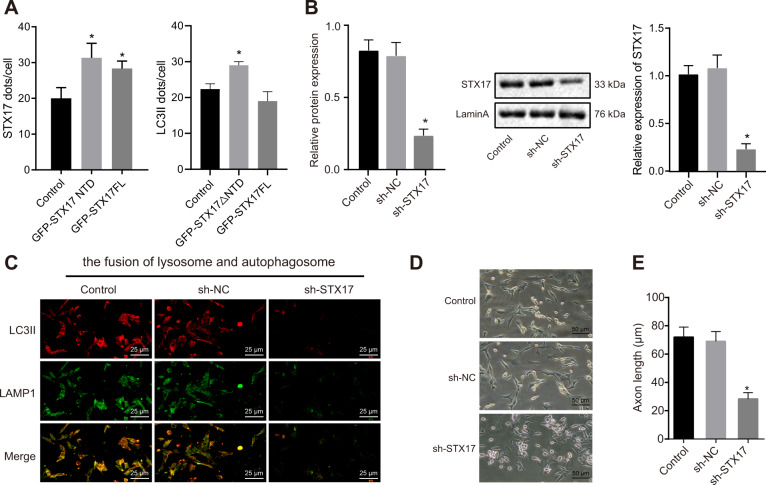
STX17 knockdown inhibits the autophagosome–lysosome fusion and reduces axon growth in HT22 cells. **A** Co-localization of LC3II and STX17 in HT22 cells without treatment, GFP-STX17△NTD-HT22 cells, or GFP-STX17FL-HT22 cells determined by immunofluorescence staining, *p < 0.05 compared with cells without treatment. The HT22 cells were treated with sh-NC or sh-STX17. **B** The silencing efficiency of STX17 determined by RT-qPCR and Western blot analysis, *p < 0.05 com*p*ared with cells treated by sh-NC. **C** The fusion of autophagosome and lysosome in cells (400×). **D** The morphological changes of axons in cells (200×). **E** The immunofluorescence staining of axons in cells detected by immunofluorescence staining, *p < 0.05 compared with cells treated by sh-NC. The experiment was repeated three times independently.

To investigate the mechanism underlying autophagosome–lysosome fusion, the HT22 cell line with GFP-STX17△NTD or GFP-STX17FL overexpression was constructed, followed by conduction of immunofluorescence staining. The results revealed that when compared with cells without treatment, the GFP-STX17△NTD-HT22 cells presented remarkably increased LC3II and STX17 expression and co-localization of LC3II and STX17. On the other hand, the GFP-STX17FL-HT22 cells showed no obvious change in LC3II expression but significantly increased STX17 (Fig. 3A). HT22 cell line with silenced STX17 was constructed, and the silencing efficiency was confirmed by RT-qPCR and Western blot analysis (Fig. 3B). Subsequently, the fusion of autophagosome and lysosome was determined. In comparison with sh-NC-treated cells, the autophagosome–lysosome fusion in sh-STX17-treated cells showed a marked decrease (Fig. 3C). Based on the morphology of axons in HT22 cells, it was found that the axon and cell body had no pronounced change in cells treated with sh-NC but the axon length and size of cell body were decreased in cells treated with sh-STX17 in relative to the cells without treatment (Fig. 3D, E and Fig. S1B). In short, STX17 silencing is able to reduce the fusion of autophagosomes and lysosomes, and may thus further affect the growth of axons.

### Knockdown of BRUCE suppresses the autophagosome–lysosome fusion by interacting with STX17

**Fig. 4:**
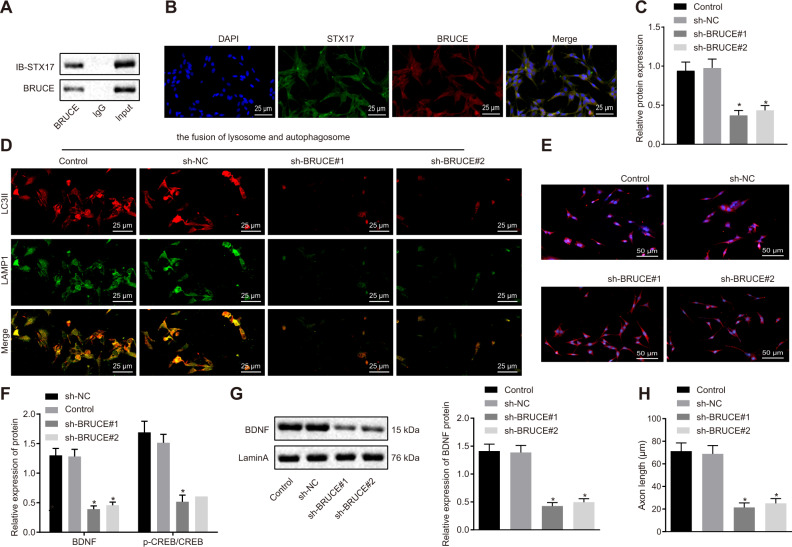
Knockdown of BRUCE represses the autophagosome-lysosome fusion and BDNF nuclear transport in HT22 cells. **A** The interaction between BRUCE and STX17 in cells determined by Co-IP assay. **B** The co-localization between BRUCE and STX17 detected by immunofluorescence staining (400×). **C** The HT22 cells treated with knocked-down BRUCE detected by Western blot analysis, *p < 0.05 compared with cells treated by sh-NC. The HT22 cells were treated with sh-NC or sh-BRUCE. **D** The autophagosome-lysosome fusion in cells (400×). **E** The BDNF nuclear transport determined by immunofluorescence staining (200×). **F** The protein expression of BDNF and p-CREB/CREB in cells, * *p* < 0.05 com*p*ared with cells treated with sh-NC. **G** The expression of BDNF in the nucleus of cells detected by Western blot analysis, *p* < 0.05 compared with cells treated with sh-NC. **H** The immunofluorescence staining of axons in cells detected by immunofluorescence staining, *p < 0.05 compared with cells treated with sh-NC. The experiment was repeated three times independently.

The Co-IP assay for exploring the relationship between BRUCE and STX17 revealed an interaction between BRUCE and STX17 (Fig. 4A). The immunofluorescence staining further verified that the existence of co-localization between BRUCE and STX17 (Fig. 4B). These results demonstrated that BRUCE might interact with STX17 and regulate the fusion of autophagosome and lysosome. To validate this hypothesis, HT22 cells were treated with silenced BRUCE, followed by Western blot analysis. Results revealed that HT22 cells exhibited notably decreased BRUCE protein expression, confirming the successful silencing (Fig. 4C). Immunofluorescence staining was performed to detect the fusion of autophagosome and lysosome, and it was found that when compared with cells without treatment, cells treated with sh-NC showed no marked change in autophagosome–lysosome fusion. In comparison with cells treated with sh-NC, the cells treated with sh-BRUCE reduced autophagosome–lysosome fusion (Fig. 4D) as well as decreased BDNF nuclear import (Fig. 4E). In addition, the results from Western blot analysis revealed that when compared with the cells treated with sh-NC, the protein expression of BDNF/TrkB signaling pathway-related factors and BDNF protein expression in the nucleus were dramatically decreased in cells transfected with sh-BRUCE (Fig. 4F, G). Also, the axon length and size of the cell body were also reduced in cells treated with sh-BRUCE relative to cells treated with sh-NC (Fig. 4H and Fig. S1C). Therefore, the interaction between BRUCE and STX17 can affect the nuclear transport of BDNF, thereby regulating the fusion of autophagosomes and lysosomes.

### miR-204 causes axonal dystrophy in AD by decreasing BRUCE

**Fig. 5:**
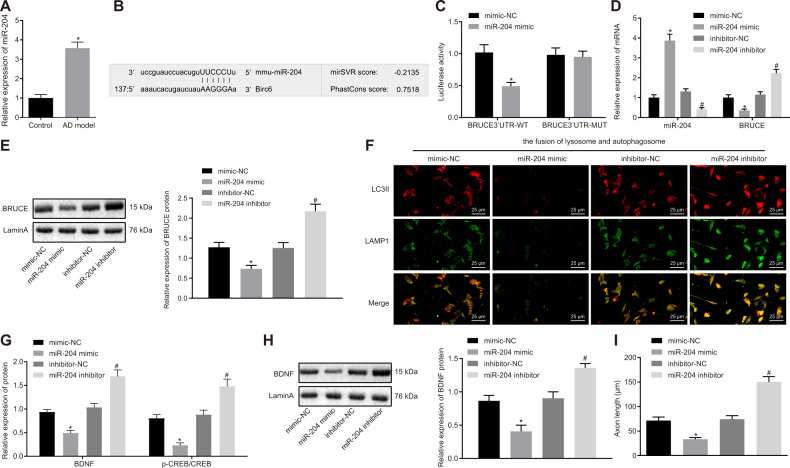
Inhibition of miR-204 promotes the autophagosome–lysosome fusion and rescues defective axonal transport by increasing BRUCE in HT22 cells. **A** The miR-204 expression in the hippocampus tissues of AD mice and normal mice, *p < 0.05 compared with normal mice. **B** The binding site between miR-204 and BRUCE3′UTR predicted by bioinformatics analysis. **C** The targeting relationship between miR-204 and BRUCE identified by dual-luciferase reporter gene assay. *p < 0.05 com*p*ared with mimic-NC treatment. The cells were treated with mimic-NC, miR-204 mimic, inhibitor-NC, and miR-204 inhibitor. **D** The expression of miR-204 and mRNA expression of BRUCE in cells measured by RT-qPCR. **E** The protein expression of BRUCE in cells examined by Western blot analysis. **F** The fusion of autophagosome and lysosome in cells detected by immunofluorescence staining (400×). **G** The protein expression of BDNF and p-CREB/CREB in cells determined by Western blot analysis. **H** The expression of BDNF protein in the nucleus of cells determined by Western blot analysis. **I** The immunofluorescence staining of axons in HT22 cells detected by immunofluorescence staining. *p < 0.05 compared with cells treated with mimic-NC; ^#^*p* < 0.05 compared with cells treated with inhibitor-NC. The experiment was repeated three times independently.

Furthermore, RT-qPCR was conducted to detect the expression of miR-204 in the hippocampus tissues of mice. Results revealed that miR-204 was obviously upregulated in the hippocampus tissues in AD mice when compared with normal mice (Fig. 5A). In silico analysis revealed that there was a binding site between miR-204 and BRUCE 3′UTR (Fig. 5B), and dual-luciferase reporter gene assay further identified that miR-204 could effectively inhibit BRUCE (Fig. 5C). HT22 cells were transfected with miR-204-mimic/inhibitor and their controls, followed by RT-qPCR and Western blot analysis to examine the mRNA and protein expression of BRUCE. In comparison with cells treated with mimic-NC, cells treated with miR-204 mimic showed significantly reduced mRNA and protein expression of BRUCE, while the cells treated with miR-204 inhibitor exhibited obviously increased mRNA and protein expression of BRUCE in relative to cells treated with inhibitor-NC (Fig. 5D, E). The immunofluorescence staining was conducted to detect the fusion of autophagosome and lysosome. Results revealed that the autophagosome–lysosome fusion was reduced in cells treated with miR-204 mimic but obviously enhanced in cells treated with miR-204 inhibitor (Fig. 5F). Next, results from the Western blot analysis showed that miR-204 mimic treatment resulted in a significant reduction in protein expression of BCNF/TrkB signaling pathway-related factors while miR-204 inhibitor treatment showed an opposite result (Fig. 5G, H). Based on the morphology of axons in HT22 cells, it was found that the axon length and size of the cell body were markedly decreased in cells treated with miR-204 but increased in cells treated with miR-204 inhibitor (Fig. 5I and Fig. S1D). In a nutshell, inhibition of BRUCE by miR-204 reduces the fusion of autophagosomes and lysosomes, which further leads to axonal dystrophy in AD mice.

### Overexpression of BRUCE rescues miR-204-triggered axonal dystrophy in HT22 cells

**Fig. 6:**
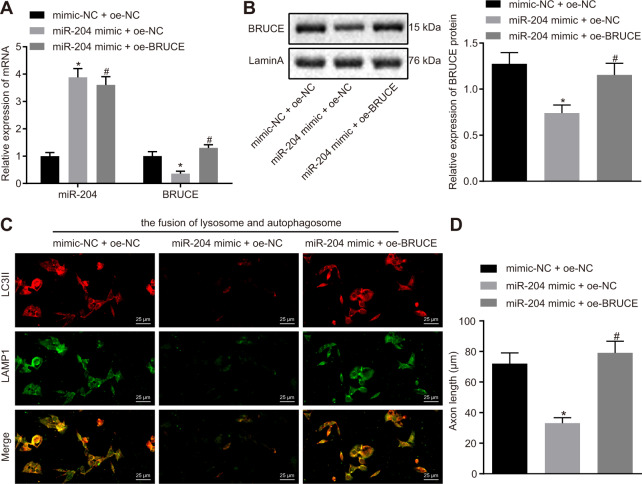
Overexpression of BRUCE rescues axons in HT22 cells. The HT22 cells were treated with both mimic-NC and o-NC, both miR-204 mimic and oe-NC, and both miR-204 mimic and oe-BRUCE. **A** The miR-204 expression and mRNA expression of BRUCE in cells detected by RT-qPCR. **B** The protein expression of BRUCE in cells detected by Western blot analysis. **C** The fusion of autophagosome and lysosome in cells detected by immunofluorescence staining (400×). **D** The immunofluorescence staining of axons in HT22 cells detected by immunofluorescence staining. *p < 0.05 compared with cells treated with both mimic-NC ad oe-NC; ^#^*p* < 0.05 compared with cells treated with both miR-204 mimic and oe-NC. The experiment was repeated three times independently.

In order to verify the effects of miR-204 and BRUCE in dystrophic axons of HT22 cells, experiments with overexpressed BRUCE were conducted. The results of RT-qPCR revealed that compared with cells treated with both mimic-NC and oe-NC, cells co-treated with miR-204 mimic and oe-NC showed a marked increase in miR-204 expression but an obvious decrease in mRNA expression of BRUCE was observed. In comparison with cells co-treated with miR-204 mimic and oe-NC, cells with combined treatment of miR-204 mimic and oe-BRUCE showed no pronounced change in miR-204 expression but showed an obvious increase in mRNA expression of BRUCE (Fig. 6A). Meanwhile, Western blot analysis was conducted to examine the protein expression of BRUCE in cells. It was found that protein expression of BRUCE was notably reduced when combined treated with miR-204 mimic and oe-NC but was obviously elevated after co-treated with miR-204 mimic and oe-BRUCE (Fig. 6B). Subsequently, immunofluorescence staining was performed to detect the fusion of autophagosome and lysosome. Results revealed that the combined treatment of miR-204 mimic and oe-NC resulted in markedly repressed autophagosome–lysosome fusion, while co-treatment of miR-204 mimic and oe-BRUCE led to significantly enhanced autophagosome-lysosome fusion (Fig. 6C). Furthermore, results from the morphological detection for the axons of HT22 cells showed that the axon length and size of cell body were reduced in cells treated with both miR-204 mimic and oe-NC, but showed an opposite result in cells treated with both miR-204 mimic and oe-BRUCE (Fig. 6D and Fig. S1E). According to the above results, the promoting effect of miR-204 on AD axonal dystrophy can be eliminated by BRUCE overexpression.

## Discussion

Axonal dystrophy, as one of the early pathological characteristics of neurodegenerative diseases including AD, is potentially causative in vesicular trafficking impairment, neuronal damage, and/or mortality^18^. It has been reported in a former study that the microtubule-stabilizing agent, Epothilone D could alleviate the disorders including axonal dysfunction and AD-like pathology in aged Tau transgenic mice^19^. However, the specific mechanism underlying the dystrophic axons in AD is still not well-characterized, thus more investigations are required to develop effective therapies for AD treatment. Most importantly, it has been demonstrated that functional-impaired crucial secretory peptidic cargos including BDNF were involved in the development of AD^20^. STX17 was evolutionarily conserved in the fusion of autophagosome and lysosomes^21^, while BRUCE was reported to participate in the regulation of autophagosome-lysosome fusion^22^. miR-204 was also reported to be involved in the axonal guidance where the abnormal expression of miR-204 was observed in mesial temporal lobe epilepsy^23^. Based on these findings, we hypothesized that the BDNF/BRUCE/STX17/miR-204 axis affects the development of axonal dystrophy in AD via regulation of autophagosome–lysosome fusion. Eventually, the findings from the present study demonstrated that silencing of miR-204 contributed to the promotion of BRUCE, and resulted in enhanced STX17-mediated autophagosome–lysosome fusion, thus improving the BDNF nuclear transport in neurons.

The results obtained from the present study revealed that the intranuclear expression of BDNF and the expression of BDNF/TrkB signaling pathway-related factors (p-TrkB/TrkB and p-CREB/CREB) were reduced in the hippocampus brain area of AD mice, accompanied by swollen and shortened axons. A former study has reported that BDNF and TrkB-TK+ were downregulated in the hippocampus of patients suffering from schizophrenia and mood disorders^24^, which was consistent with our results that APP/PS1 transgenic mice present BDNF/TrkB signaling deficits. The pronounced decline in the levels of BDNF and TrkB was also found in the early stage of AD^25^. Additionally, reduced expression of BDNF exons IX and IV, and decreased CRE (BDNF) binding activity was observed in the hippocampus area of glucocorticoid receptor impaired mice^26^. Furthermore, based on the definition of axonal dystrophy, swollen and tortuous neuronal axons have induced synaptic changes in AD^27^. Therefore, the previous findings were consistent with the results of the present study, where the poorly expressed BDNF/TrkB signaling pathway was correlated with axonal dystrophy in AD.

In addition, results from the present study revealed that the induction of axonal dystrophy by repressing the autophagosome–lysosome fusion has reduced the BDNF nuclear transport. As mentioned above, autophagosome was the key component in dystrophic axons, and lysosomes were the crucial component involved in the neuronal function and mutations in genes inducing lysosome formation, as well as activity related to neurodegenerative disorders^10,28^. A previous study has been revealed that the retrograde transport of BDNF-activated TrkB receptors participated in the process of autophagosomes to promote neuronal complexity and prevented neurodegeneration in vivo^8^. The present study demonstrated that BRUCE interacted with STX17, and the silencing of both BRUCE and STX17 could reduce the autophagosome–lysosome fusion. Similarly, the interaction between BRUCE and STX17 was also proven in former research, where BRUCE modulated the STX17-mediated autophagosome–lysosome fusion^11^. Furthermore, this study revealed the upregulation of miR-204 in hippocampus tissues of AD mice as well as in HT22 cells and showed that miR-204 targeted BRUCE, thus resulting in suppressed autophagosome–lysosome fusion and dystrophic axons. However, the recovered expression of BRUCE has reversed all the effects induced by miR-204. The highly expressed miR-204 in the distal axons of sympathetic neurons has also been identified in a previous study^13^. Downregulation of miR-204 which contributed to the elevated expression of LC3II, a key biomarker of the autophagosome, in myocardial ischemia-reperfusion injury has also been reported in a previous research^29^. Most importantly, BRUCE enhanced the fusion of autophagosome and lysosome^11^. This evidence is in line with our study where the role of silenced miR-204 and overexpressed BRUCE which contributed to the promotion of autophagosome–lysosome fusion and amelioration of axonal dystrophy in AD was proven.

**Fig. 7:**
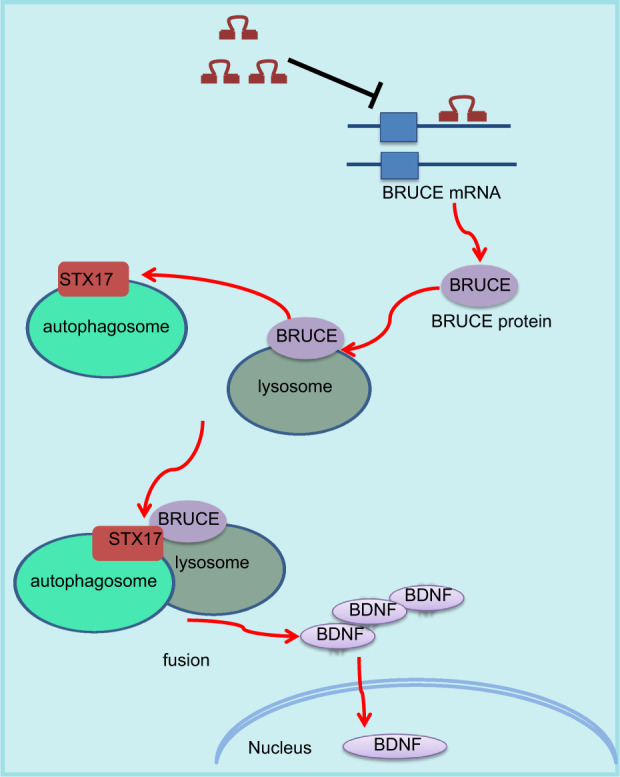
The regulatory mechanism involving miR-204/BRUCE/STX17/BDNF that affects the dystrophic axons induced by AD via autophagosome–lysosome fusion mediation. Silencing of miR-204 promoted BRUCE to enhance the STX17-mediated autophagosome-lysosome fusion, thus improving the BDNF nuclear transport and alleviating the dystrophic axons of AD mice.

In conclusion, the key findings of the present study suggest inhibition of miR-204 prevents defective axonal transport by blocking BRUCE interaction with STX17 in hippocampal neurons. This study provides new insight into novel therapeutic targets for AD treatment (Fig. 7). Nevertheless, it should be also pointed out that more in-depth investigations are still required to explore the underlying mechanism of BDNF mediating autophagosome–lysosome fusion. It is also necessary to further identify the specific interaction between miR-204 and IAP family members since their targeting relationship has been rarely reported.

## Supplementary information

Supplementary Information
