# Supplementary material for: BRUCE silencing leads to axonal dystrophy by repressing autophagosome-lysosome fusion in Alzheimer’s disease
Source: Transl Psychiatry. 2021 Aug 5;11:421. doi: 10.1038/s41398-021-01427-2 (PMC8342531; doi:10.1038/s41398-021-01427-2)
Supplement: Supplementary file 1 — Supplementary Information [file 41398_2021_1427_MOESM1_ESM.docx]

**
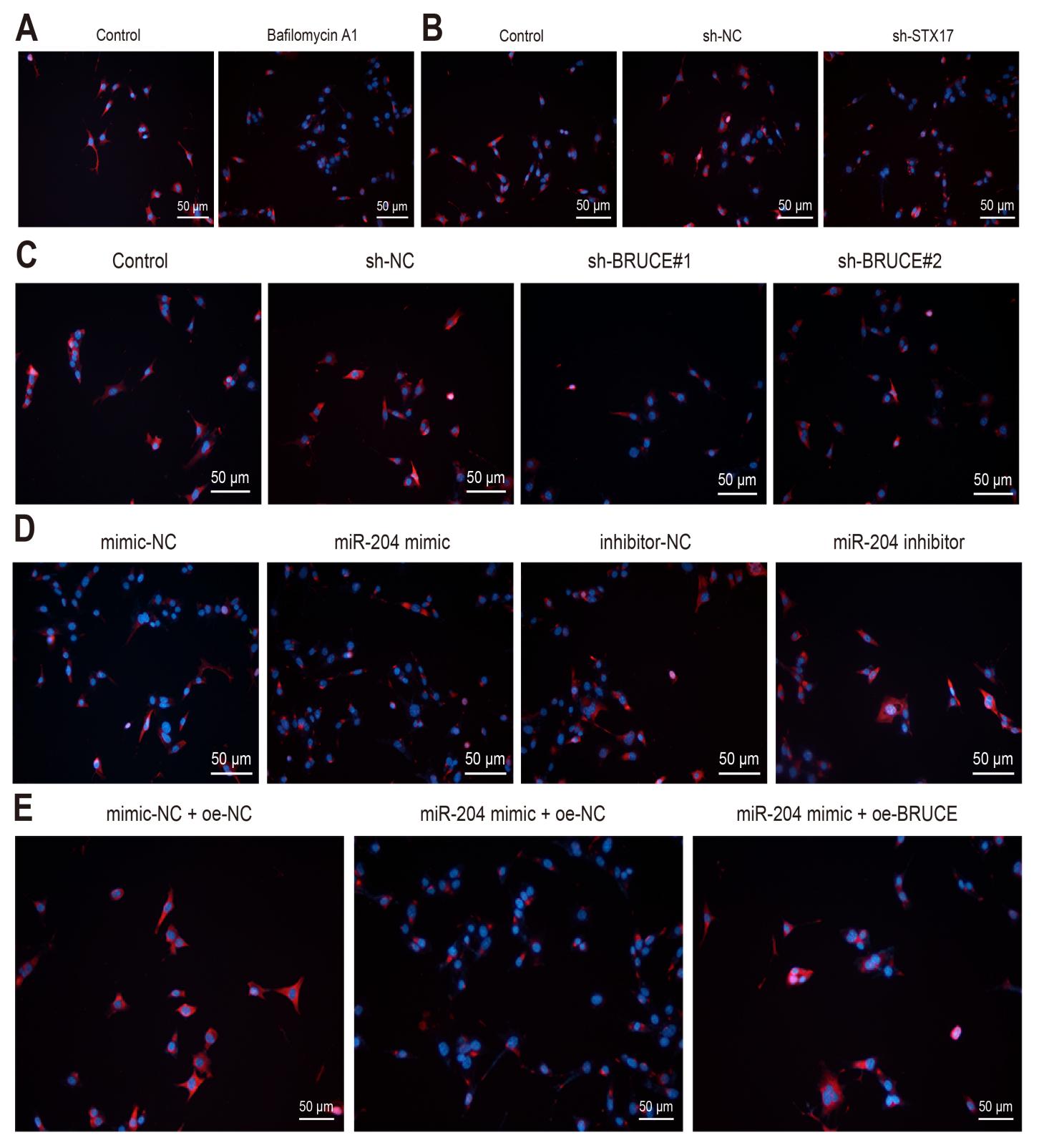
**

**Fig. S1** A, Representative image of immunofluorescence staining of axons of HT22 cells after interference with 30 nM of Bafilomycin A1 for 24 h, Tau as a marker for axons (200 ×). B, Representative image of the immunofluorescence staining of axons in cells after sh-NC or sh-STX17 treatment (200 ×). C, Representative image of the immunofluorescence staining of axons in cells after sh-NC, sh-BRUCE#1 or sh-BRUCE#2 treatment (200 ×). D, Representative image of the immunofluorescence staining of axons in cells after mimic-NC, miR-204 mimic, inhibitor-NC or miR-204 inhibitor treatment (200 ×). E, Representative image of the immunofluorescence staining of axons in cells after mimic-NC, miR-204 mimic, oe-NC or oe-BRUCE treatment (200 ×).

**Table S1** Primer sequences for RT-qPCR.

| Target | Primer sequence |
| --- | --- |
| BDNF | F: 5’-TCATACTTCGGTTGCATGAAGG-3′ |
|  | R: 5′-ACACCTGGGTAGGCCAAGTT-3′ |
| TrkB | F: 5’-CGTCACTTCGCCAGCAGTAG-3′ |
|  | R: 5′-CTGCGGTAGCAGGACAGTG-3′ |
| BRUCE | F: 5′-TCACGGGGCTTCTAGAGGTT-3′ |
|  | R: 5′-AACCCCGAAGGAACTCACAG-3' |
| CREB | F: 5′-CAGGGGTCGCAAGGATTGAA-3′ |
|  | R: 5′-CATCGCCTGAGGCAGTGTA-3' |
| miR-204 | F: 5′-TTCCCTTTGTCATCCTATGCCT-3′ |
|  | R: 5′-GTGCAGGGTCCGAGGT-3' |
| U6 | F: 5'-CTCGCTTCGGCAGCACA-3' |
|  | R: 5'-AACGCTTCACGAATTTGCGT-3' |
| GAPDH | F: 5'-AGGTCGGTGTGAACGGATTTG-3' |
|  | R: 5'-GGGGTCGTTGATGGCAACA-3' |

Note: RT-qCPR, reverse transcription quantitative polymerase chain reaction; F, forward; R, reverse; BDNF, brain-derived neurotrophic factor; TrkB, Tyrosine Kinase receptor B; BRUCE, BIR repeat containing ubiquitin-conjugating enzyme; CREB, p-cAMP-response element binding protein; miR, microRNA; GAPDH, glyceraldehyde-3-phosphate dehydrogenase.
